# Meningitis in Childhood

**Published:** 1955-09

**Authors:** Beryl D. Corner

**Affiliations:** Consultant Paediatrician, United Bristol Hospitals and Southmead Hospital


### MENINGITIS IN CHILDHOOD

### (Purulent and Tuberculous Infections)

BY

BERYL D. CORNER, M.D., F.R.C.P.
*Consultant Paediatrician, United Bristol Hospitals and Southmead Hospital*

### MENINGITIS

" The man who starts for the first time to study the diseases of children is like a traveller in a foreign country; he hears a strange language spoken which he does not understand. At all events, if the language is not absolutely strange, it is spoken with a foreign accent, for the physical signs of disease are often different in children from what they are in grown-up people, so that you have to devote special attention to their interpretation." These remarks were attributed to Dr. Charles West, founder of the Hospital for Sick Children, Great Ormond Street, by Hutchison (1944).

Acute suppurative meningitis and tuberculous meningitis are most commonly encountered in childhood so that it is proposed to consider the clinical features of these conditions in children. Data have been obtained by analysis of cases admitted to the paediatric departments of United Bristol Hospitals and Southmead Hospital over the five-year period, 1948–53.

### PURULENT MENINGITIS

A study of the literature reveals a good deal of inadequacy in description of the clinical features of this disease in its early stages and as it appears in different age groups in children. Henoch (1899), in his beautiful account of purulent meningitis which is well illustrated with case histories, describes the typical picture in the well-d veloped case as we see it in the child of school age and also mentions the high incidence of convulsions in infancy and the difficulty of diagnosis in the more chronic type of meningococcal meningitis. Reference to modern standard text-books shows a remarkable uniformity in the clinical description, " The onset of the disease is generally sudden and is characterized by vomiting, headache, sometimes pain in the back, temperature 102°–104°. There may be convulsions at the onset. From the beginning the signs of meningitis are generally evident ", etc. (Sheldon). This is a description of meningococcal meningitis which is familiar in the adult and in the older ages of childhood, but in the earliest years the language may be strange, the accent different. Hence, only too frequently is still seen the tragedy of the young child, who is sent into hospital so late in the disease, that the most effective drugs available are useless against the brain damage which is irreversible, and will either cause death or the survival of a useless individual.

In the past, a policy of procrastination before taking action in undiagnosed acute illness in early life gave little cause for concern as treatment was rarely effectual. Today treatment of the early case of acute purulent meningitis is so dramatic and rewarding with the antibiotic drugs that it is imperative that if a suspicion of this diagnosis crosses the doctor's mind immediate steps should be taken to get this confirmed by lumbar puncture, so that treatment may be started at the earliest possible moment.

In attempting to elucidate the problems of early clinical diagnosis, the records of 105 patients have been studied with particular reference to the history before admission to hospital and the important physical features on examination. These patients fell into four age groups, the disease in each of which seemed to present certain characteristic features which differed from those at other ages. Tables 1 and 2 and 3 show the number of patients in each group, the mortality rate, and the type of organism causing the infection.

The high incidence of the disease (57 per cent of all cases) and the greater mortality rate in children under one year is at once apparent, but it should not be assumed that this age distribution is necessarily that always found in the general population, as owing to the special facilities for infant care at the hospitals concerned in this survey, a high proportion of young children with undiagnosed acute illness tends to be admitted to them.

The organism most frequently concerned is seen to be the *meningococcus*, but the low mortality rate with modern treatment in early cases is particularly notable. The only death occurred in a six-months-old infant who had been ill for twenty-four hours, and on admission was moribund due to fulminating meningococcal septicaemia which had caused suprarenal haemorrhage, the Waterhouse-Friderichsen syndrome.

*Haemophilus influenzae* commonly causes meningeal infection in infancy and in this series there were 2 deaths in 26 patients, which compared favourably with Alexander's (1947) result of 3 deaths in 25 patients. Pneumococcal meningitis carried the highest mortality rate which paralleled the findings of other authors, but in this series it seemed that these cases were particularly liable to be labelled "pneumonia" until the child became unconscious and beyond all hope of successful treatment.

There was a small number of cases in whom the cerebrospinal fluid showed changes typical of acute purulent meningitis but no organisms could be cultured. Some of these cases occurred early in the series when bacteriological methods were perhaps not so efficient as now and, in others, antibiotics had been given before admission.

It should be remembered that the symptoms and signs of purulent meningitis are produced by: (1) Bacterial toxaemia, (2) Cerebral irritation due to the presence of inflamed meninges and increased pressure of purulent cerebrospinal fluid and (3) Cerebral compression with cerebral oedema and sometimes vascular lesions in the brain. The dominance of any one of these factors gives us the varying clinical picture as seen in individual cases.

The clinical features in four age groups will now be considered. It is not proposed to ascribe clinical differences to the various bacteriological types of purulent meningitis, as apart from the haemorrhagic tendency in meningococcal infection, there were no very clear-cut distinguishing features in the cases analysed.

*Neonatal Meningitis* (*Table* 4)

There were fourteen children who developed purulent meningitis in the first few weeks of life. As in many infections of the newborn infant, the causative organism in seven cases was *B. coli*; the meningeal infection being the terminal phase of a septicaemia which arises from a local lesion in the renal tract, liver or lungs. This is a particularly fatal type of meningitis since, until chloramphenicol became available, treatment was usually unsuccessful. The only surviving case in this series was treated with that drug.

Nine children were less than ten days old, and in five of these the infection was probably directly related to problems of labour and delivery. In two cases, early rupture of the membranes followed by infection of the liquor amnii, produced antenatal infection of the infant, which might have been avoided by the administration of suitable antibiotics to the mother, when her membranes had been ruptured for twenty-four hours without the onset of labour. One infant, who died at the age of 21 days from *B. coli* meningitis, developed septicaemia three days after an abrupt change from full breast feeding to full cream dried milk. The disturbance caused by this sudden change in the bacterial flora of the upper intestinal tract may well have been responsible. In addition to the cases analysed, there were several infants with serious congenital abnormality of the central nervous system, e.g. meningo-myelocele, who developed terminal meningitis. These have been deliberately omitted from this analysis.

The pattern of symptoms in this age group is apparent from the table. The usual features are a change in colour of the skin, so that the child looks pale with a tendency to episodes of cyanosis, fretfulness, anorexia and irritability. Unexplained fever may be noticed, or occasionally the temperature is subnormal. Examination revealed an infant whose greyish pallor was usually striking, but in whom no other physical signs could be found, although occasionally abnormal jerking of the limbs or rigidity were seen. In only five patients was abnormality of the anterior fontanelle evident, and neck rigidity was absent in all but three. Kernig's sign was invariably absent.

In the neonatal period the possibility of meningitis, or some other intracranial catastrophe, may be so easily overlooked that lumbar puncture is now one of the most commonly performed diagnostic procedures, and should never be omitted, or delayed if no other satisfactory diagnosis can be made to account for the child's symptoms. The only four children who survived in this series were diagnosed very early in the disease.

*Infancy. One month to one year* (*Table* 5)

At this age cerebral irritation was the predominant feature as a rule. Refusal to feed preceded all other symptoms and was often followed by irritability. A common history was that the baby slept fitfully, awakened frequently with a miserable but feeble cry, and in the words of one mother " whimpered like a puppy ". The high-pitched shriek often considered characteristic of meningitis was rarely noticed.

A very characteristic symptom commented on by nearly all parents was the child's dislike of being touched or nursed. This point requires particular emphasis as most sick children are comforted in their mother's arms, but the baby with early meningitis prefers to be left alone. Vomiting may be an early symptom but is not infrequently absent throughout the whole of the disease and in this series only twenty-two of the forty babies vomited before admission. Diarrhoea and signs suggestive of abdominal pain were occasionally present, when the child had attacks in which it screamed and drew its legs up; close observation sometimes revealed that this attack was actually a convulsion. Photophobia may occur but was only noticed once in this series of cases.

*The second type* of clinical picture is characterized by sudden lethargy and limpness which is due either to cerebral compression, or the overwhelming bacterial infection that, in meningococcal meningitis particularly, may give rise to haemorrhages and acute suprarenal insufficiency. Most mothers commented on the striking pallor of the baby and a few noted alterations in the respiration, such as grunting, rapid or irregular breathing, which sometimes was so pronounced that a diagnosis of respiratory disease was made. If convulsions occur they are a helpful diagnostic symptom but are rarely the earliest evidence of meningitis and Ounsted (1951) showed that convulsions were of serious portent. One baby was found in coma before the mother was aware of any illness.

A history of upper respiratory infection within the preceeding three weeks was found in almost every case irrespective of the type of clinical onset or the causative organism.

*Clinical Examination.* The diagnosis can be made or suspected almost entirely by inspection of the child, who should be examined in a good light and preferably unclothed. The posture and response to handling by the mother should first be noted; the baby usually lies still and limp but is sometimes restless and irritable, even so these children are usually too ill to put up any resistance to physical examination which is a helpful point in differential diagnosis.

The head should always be palpated and if the anterior fontanelle is still open, increased tension may be noted. This is however a variable sign, the tension being normal in many of our cases. The face is usually characteristic being pale with an expression of pain. The eyes tend to have a " far-away " look or may move unnaturally, sometimes there is photophobia and rarely a squint. Peculiar sucking and twitching movements of the mouth may be noted, and respirations are often grunting and rapid, or sometimes irregular or periodic. The pulse rate is variable, and in the comatose patients may be slowed due to cerebral compression. Temperature also shows great variation, being occasionally at hyperpyrexial levels, or in the shocked fulminating cases, sub-normal. Neck and generalized rigidity of the trunk and limbs is sometimes present but was absent in many of the patients of this series, particularly in the younger age groups, and Kernig's sign is more often absent than present in infancy. A careful search of the skin may reveal a few petechiae or purpuric patches, but even in cases of meningococcal infection less than half of the children had a rash at any time.

*Table* 6. shows the four most important features noted on admission of the patient to hospital. The frequency of the finding of skin pallor may help the doctor to consider meningitis as a possible cause of the baby's illness, rather than some of the common but less serious infective illnesses of infancy, which more often cause flushed cheeks. It will also be seen that there was no meningismus in almost one-third of the cases at this age, and that a quarter were admitted in convulsions. In view of the frequency of convulsions at some stage of the disease in young children, it should be the rule that a convulsion occurring for the first time in a child with fever is an indication for lumbar puncture.

*Older Age Groups*

The clinical text-book picture is seen with greater frequency as infancy is left behind. The " one- to three-year-old " group of children was still very liable to convulsions in the early stage of the illness, but only one child over the age of three years had a fit. As in infancy, most of the children appeared pale and shocked when admitted to hospital and three-fifths of them were in coma or stupor, which demonstrated the deadly rapidity with which these diseases overwhelm the child before its nature is suspected by either parents or doctors. Over three years of age, the classical triad of headache, vomiting and prostration was present in every patient. As a rule in this group diagnosis presented little real difficulty.

*Cause of Death* (*Table* 7)

It is profitable to examine the reasons for the deaths in this series. Omitting the special group of neonatal cases, it will be seen that all these patients with one exception were infants. The salient features are the long history of symptoms before admission in six cases, all of whom had been labelled " influenza "; in two cases the child had been ill for fourteen days. These six cases were admitted in deep coma, with signs of shock. Eight deaths occurred within a few hours of admission. Two grossly abnormal children developed terminal meningitis. Lumbar puncture was performed in every case within a few minutes of admission and except in cases 11 and 12, intensive treatment was begun as soon as the purulent cerebrospinal fluid was seen. The two cases who died later than twenty-four hours after admission remained in coma with convulsions throughout that period, and showed little response to treatment although various antibiotics were tried according to the drug sensitivity of the organism. These cases serve to underline the tragedy of late diagnosis in this disease.

### TUBERCULOUS MENINGITIS (TABLE 8)

The material comprised 84 cases of whom 68 were treated (cases still under treatment have been excluded). The remainder were considered too advanced to benefit at the time that streptomycin was in short supply. The survival rate was 65 per cent for treated cases. The classification of cases is that used by the Medical Research Council, as follows:

1. *Early* (*mild*). Patients with non-specific symptoms, little or no clinical signs of meningitis, no paresis, in good general condition and fully conscious.

2. *Medium* (*or moderate*). All cases between grades 1–3, who were conscious but often irritable, and with meningeal signs present.

3. *Advanced* (*severe*). Patients obviously severely ill, deeply stuporose or comatose or with severe paresis.

*Symptoms and Physical Signs*

Table 9 shows all the symptoms noted prior to hospital admission. It will be seen that four children were known to have had turberculous lesions previously, a severe blow on the head preceded symptoms in four others by one to four weeks, and erythema nodosum had occurred in two cases within a few weeks of the onset of symptoms.

The first symptom was always a change in the usual behaviour of the child. The school-age child became listless, wanted to sit continually, refused to go to school or out to play. The toddler often became much less active, lay about and fell asleep unexpectedly during the day and was wakeful and fretful at night. During this first stage abdominal symptoms were common. There was vomiting in 82 per cent of cases, it was often occasional and not related to food, but tended to occur mostly during the daytime when the child moved about, rather than in bed at night. Sometimes persistent vomiting was the only definite symptom. The appetite was always capricious and the bowels usually constipated. Abdominal pain was the principal symptom in 18 of the 84 cases, so that this diagnosis should always be considered in the differential diagnosis of obscure abdominal pains of sudden onset in childhood. Diarrhoea occasionally accompanied the pain and ketosis was often present.

The temperature had been taken in only 25 per cent of the children and in all these it was found to be raised at some time during the day, usually to 100°–101°. Sixteen children had fits prior to admission, but in most of these the fit heralded a later stage of the disease and was never a very early symptom. Cough was present in a few cases; in seven children there had been a sudden onset of paralysis but, like convulsions, this always occurred in the second or third stage. In taking the history of such cases, a search for possible tuberculous contact, which was often only obtained on direct questioning, was helpful in arousing suspicion of this disease.

*Clinical Examination.* In the first stage of the disease the child was usually rather limp and quiet, but perhaps slightly miserable and irritable, often with a sallow skin and flushed cheeks. Wasting at this stage was moderate or absent. Frequent sighing was often noted and the pupils tended to remain dilated. Temperature or pulse were always slightly raised. It should be emphasized that there were no other signs of meningeal disease.

When the child reached the second stage, irritability was greater, but consciousness was preserved and typical signs of mild meningismus were present. In contrast to purulent meningitis neck stiffness was slight. The child tended to lie very still and often curled up. Diagnosis was now usually obvious.

The third stage cases were all stuporose or restless and mentally confused. In most cases speech had gone, wasting was severe and the child was febrile, with marked meningismus and incontinent. Often it was unable to swallow and occasionally paralysis of the face, oculomotor muscles or limbs was present.

*Results of Treatment*

Although it is outside the scope of this paper to discuss treatment, it should be emphasized that the results of treatment in this disease depend so much on the diagnosis in the first or early second stage. In the Ministry of Health Streptomycin Trials, the overall survival rate was 28·6 per cent but in early cases this figure rose to 56·5 per cent. In our much smaller series, only one early case and seven second-stage cases died and survival rates of 86–90 per cent have been reported more recently by Mackay (1954) Manchester and Lober (1954) Sheffield.

### SEQUELAE OF MENINGITIS

Unfortunate sequelae were occasionally seen in cases who recover from both purulent and tuberculous meningitis (see Table 10). In the series of cases of purulent meningitis, eleven children show some after-effects, all of whom were infants or children under three years of age. One child died from resulting hydrocephalus six months after infection was cured. Two children have been left with severe cerebral palsy and mental impairment, and two others have slight residual spastic hemiplegia. Two have developed epilepsy and two are severely deaf but otherwise normal; mild squints are present in two children.

The patients who survived tuberculous meningitis have now been followed up for several years, twelve of these cases started treatment in 1947 and on the whole the long-term results are very gratifying. Only three cases relapsed after apparent cure. An attack of measles precipitated the relapse in two, one of whom unfortunately died quite rapidly, the other two were successfully treated again and have remained well and free from sequelae.

Mild residual spastic hemiplegia is present in one child but is not severe enough to interfere with normal activity, and one child has a unilateral lower facial paralysis. Deafness has been the most serious problem, five children who were treated with dihydrostreptomycin have become severely deaf, one child moderately and another slightly deaf. This drug has since been discontinued for treatment.

As far as has been ascertained up to date the mental capacity of the forty-four recovered children does not seem to have been affected. Several girls are now employed in full-time clerical work, one boy is in the merchant navy, two girls have gained grammar school scholarships and another has won a free place to the Mary Haire Grammar School for the partially deaf.

Since the discovery of antibiotics a revolution has taken place so that these diseases, which only twenty years ago were almost untreatable, now have mortality rates of less than 20 per cent. Even these deaths are a reproach to our profession and a spur to greater effort, since by earlier diagnosis it should be possible to reduce them to negligible figures.

It is popular to believe that for advance in diagnosis an elaborate paraphernalia of apparatus and tests is necessary, but in meningitis, as in many other diseases, the secret of success lies still in the fundamental skill of clinical observation which every doctor has in his power to use, the searching eye to look at the child's face, the listening ear to hear its cry and the mother's story, and the sensitive figure to palpate the fontanelle.

I should like to acknowledge with grateful thanks the help of all those physicians on the staff of United Bristol Hospitals and Southmead Hospital who have allowed me to examine the records of their cases for the purpose of this paper and particularly to Professor Perry, Professor Neale and Dr. John Apley.

### REFERENCES

Hutchinson, R. and Moncrieff, A. (1944): *Lecture on Diseases of Children*, ninth edition, p. 3. London.

Henoch, E. (1889): *Lectures on Children's Diseases*, Vol. 1, translated from fourth edition by John Thompson, pp. 336–349. London.

Sheldon, W. (1946): *Diseases of Infancy and Childhood*, p. 502. London.

Alexander, H. E. (1947): *Advances in Paediatrics*, Vol. 11, p. 129. New York.

Ounsted, C. (1951): *Lancet*, Vol. CCLX, p. 1245.

Mackay, R. I. (1954): *Archives of Diseases in Childhood*, Vol. 29, p. 366.

Lorber, J. (1954): *Archives of Diseases in Childhood*, Vol. 29, p. 366.

TABLE 1

**Purulent Meningitis**

| | | |
|---|---|---|
| *Total cases* . . . . . . | 105 | |
| First month . . . . . . | 14 cases | 10 deaths |
| $\frac{1}{12}$–1 year . . . . . . | 46 ,, | 8 ,, |
| 1–3 years . . . . . . | 24 ,, | 3 ,, |
| 3–14 years . . . . . . | 21 ,, | 1 ,, |

TABLE 2

**Types of Meningitis (age 1 month to 14 years)**

| Organism: | Cases | Died |
|---|---|---|
| Meningococcus . . . . | 37 | 1 |
| *H. influenzae* . . . . | 26 | 2 |
| Pneumococcus . . . . | 15 | 6 |
| *B. coli* . . . . . . . . | 2 | 2 |
| Streptococci . . . . . . | 1 | — |
| Staphylococci . . . . . . | 2 | 1 |
| Unidentified . . . . . . | 8 | — |

TABLE 3

**Type of Meningitis—Age Distribution**

| Age | Meningo-coccus | | *H. influenzae* | | Pneumo-coccus | | *B. coli* | | Strepto-cocci | | Staphylo-cocci | | Un-iden-tified |
|---|---|---|---|---|---|---|---|---|---|---|---|---|---|
| | Total | Died | Total | Died | Total | Died | Total | Died | Total | Died | Total | Died | Total |
| First month | — | — | — | — | — | 1 | 7 | 6 | 4 | 2 | — | 1 | 1 |
| 1 month to 1 year . . | 21 | 1 | 8 | 1 | 8 | 3 | 2 | 2 | — | — | 2 | 1 | 5 |
| 1–3 years . . | 11 | — | 10 | 1 | 3 | 2 | — | — | — | — | — | — | — |
| 3–14 years . . | 5 | — | 8 | — | 4 | 1 | — | — | 1 | — | — | — | 3 |

TABLE 4

### Neonatal Meningitis

| Case | Age (in days) and type of delivery | First symptoms | Signs | Organism | Result |
|---|---|---|---|---|---|
| 1 | 11 Forceps | Fretfulness | Pyrexia | Purulent C.S.F. culture, sterile | Lived: no sequelae |
| 2 | 6 Normal | Incessant whining, intermittent cyanosis | Grey pallor, bulging fontanelle, pyrexia | Streptococcus | Lived: residual squint, normal otherwise |
| 3 | 9 Premature | Refusal to feed, pyrexia | Very irritable, rigid | *B. coli* | Lived: no sequelae |
| 4 | 28 | Off food, pyrexia | Convulsions, fontanelle full | *Streptococcus viridans* | Lived: normal |
| 5 | 9 Breech, premature | Lethargy, pallor, cyanotic attacks | Pyrexia | B. haemolytic streptococcus | Died |
| 6 | 7 Breech | Lethargy, subnormal temperature | Grunting respirations, bulging fontanelle | *B. coli* | Died |
| 7 | 5 Early, ruptured membranes | Irritability, subnormal temperature | Rigidity | *B. coli* | Died |
| 8 | 4 Normal | Cyanotic attacks, pyrexia | Slight head retraction | *B. coli* | Died |
| 9 | 5 Normal | Cyanotic attacks, pyrexia | Full fontanelle, grunting respirations | *Streptococcus agalactiae* | Died |
| 10 | 28 | Anorexia, coryza | Pyrexia, twitching | Pneumococci | Died |
| 11 | 21 | Screaming, drawing legs up, refusal to feed | Pallor, dehydration | *B. coli* | Died |
| 12 | 21 Premature | Irritability, rigidity | Pallor, bulging fontanelle | *Staphyloccus aureus* | Died |
| 13 | 2 Membranes ruptured 1 week | Cyanotic attacks, pyrexia | Grey, limp | *B. coli* | Died |
| 14 | 2 Premature, forceps | Irritability, sudden collapse | Respiratory irregularity, grey | *B. coli* | Died |

TABLE 5

**Purulent Meningitis—Symptoms before Admission**
(Figures refer to number of cases)

| | 1 month to 1 year | 1–3 years | 3–14 years |
|---|---|---|---|
| 1. Irritability and fretfulness .. .. | 24 | 10 | 1 |
| 2. Vomiting .. .. .. .. | 22 | 14 | 16 |
| 3. Anorexia .. .. .. .. | 24 | 7 | 5 |
| 4. Lethargy .. .. .. .. | 17 | 13 | 10 |
| 5. Headache .. .. .. .. | — | — | 13 |
| 6. Convulsions .. .. .. .. | 9 | 8 | 3 |
| 7. Rash .. .. .. .. .. | 9 | 4 | 4 |
| 8. Pyrexia .. .. .. .. | 3 | 6 | 5 |
| 9. Coma .. .. .. .. .. | 1 | 2 | — |
| 10. Altered respirations .. .. | 3 | 1 | — |
| 11. Other .. .. .. .. .. | 2 | — | 2 |
| Total cases .. .. .. | 46 | 24 | 21 |

TABLE 6

**Purulent Meningitis—Condition on Admission**

| Signs | 1 month to 1 year | 1–3 years | 3–14 years |
|---|---|---|---|
| Pallor .. .. .. .. .. | 33 | 17 | 13 |
| Meningismus .. .. .. .. | 30 | 20 | 21 |
| Stupor or coma.. .. .. .. | 20 | 14 | 13 |
| Convulsions or twitching .. .. | 12 | 2 | 2 |
| Total cases .. .. .. | 46 | 24 | 21 |

TABLE 7

**Purulent Meningitis—Deaths**

| No. and age | Organism | History | Admission | Remarks |
|---|---|---|---|---|
| 1. 6 months | Meningo-coccus | 20 hours. Vomiting and listless | Coma. Severe shock | Died 7 hours after admission. Supra-renal haemorrhages |
| 2. 6 months | *H. influenzae* | 14 days. Fever. Intermittent vomiting. Crying + Coryza | Coma. Convulsions. Neck rigidity | Died 4 days after admission |
| 3. 2 years 8 months | *H. influenzae* | 7 days. Coryza. Staring. Refused food. Only vomited once. | Coma. Severe shock and dehydration | Died 6 hours after admission |
| 4. 3 months | Pneumo-coccus | ? 7 days. Anorexia. Vomit. Whimper-ing. Coryza | Moribund | Died just after admission |
| 5. 7 weeks | Pneumo-coccus | Vomit. Anorexia. Pallor. 2 days | Convulsing +++ Grey. No neck rigidity. Fontanelle *not* + | Died 6 hours after admission |
| 6. 10 months | Pneumo-coccus | 2 days. Anorexia Grunting. | Pallor — Semi-conscious | Died 12 days after admission |
| 7. 14 months | Pneumo-coccus | 14 days. Irritable and Fever. Convulsions | Coma. Shock. Neck stiff. Convulsing | Died 6 hours after admission |
| 8. 14 months | Pneumo-coccus | 5 days. Irritable 2 days. Anorexia and drowsy | Coma. Twitching. *No* stiff neck. Respirations irregular | Died 12 hours after admission |
| 9. 5½ years | Pneumo-coccus | 3 days. Head injury. Headache. Vomiting. Convulsions | Convulsing. Temp. 105°. Stiff neck | Died 18 hours after admission |
| 10. 7 weeks | *B. coli* | 3 weeks. Limp and staring. Convulsions and vomiting 3 days | Moribund. Bulging fontanelle | Died few hours after admission |
| 11. 6 weeks | *B. coli* | Rising respiration rate and later convulsions | Fontanelle tense. Neck rigidity. Greyish pallor | Meningitis was a terminal event in a grossly abnormal baby with multiple congenital abnor-malities including imperforate anus |
| 12. 11 months | *Staphylo-coccus aureus* | Refusal of food. Vomiting. Lethargy | Moribund | Subdural haema-toma since early infancy. Grossly re-tarded child men-tally. Operation was performed 4 weeks previously |

TABLE 8

**Tuberculous Meningitis**

| | |
|---|---|
| Total Cases .. .. .. | 84 |
| Recovered .. .. .. | 44 |
| Died (Treated) .. .. .. | 24 |
| Died (Untreated).. .. .. | 16 |

TYPE OF CASE (TREATED)

| | Early | | Medium | | Advanced | |
|---|---|---|---|---|---|---|
| | Lived | Died | Lived | Died | Lived | Died |
| Under 1 year .. | 2 | — | — | 2 | 3 | 1 |
| 1–3 years .. .. | 2 | — | 6 | 4 | 3 | 5 |
| Over 3 years .. | 7 | 1 | 18 | 1 | 3 | 10 |
| Total cases .. | 11 | 1 | 24 | 7 | 9 | 16 |

TABLE 9

**Tuberculous Meningitis**

Total Cases: 84

Symptoms before Admission:

| | |
|---|---|
| Vomiting .. .. .. | 69 |
| Lethargy .. .. .. | 58 |
| Anorexia .. .. .. | 33 |
| Headache .. .. .. | 32 |
| Abdominal pain .. .. | 18 |
| Pyrexia .. .. .. .. | 21 |
| Fits .. .. .. .. | 16 |
| Cough .. .. .. .. | 17 |
| Paralysis .. .. .. | 7 (4 eyes, 2 arms, 1 face) |
| Other T.B. lesions .. .. | 4 |
| Head-injury history .. .. | 4 |
| Erythema nodosum .. .. | 2 |

TABLE 10

**Sequelae**

PURULENT MENINGITIS (11 PATIENTS)

| | Age of onset: | Type of meningitis: | Residual signs: |
|---|---|---|---|
| 1. | 5 days | Streptococcal | Mild strabismus |
| 2. | 6 weeks | Meningococcal | Severe hydrocephalus; died later |
| 3. | 11 months | Meningococcal | Occasional major fits |
| 4. | 11 months | Meningococcal | Mild spastic hemiplegia |
| 5. | 15 months | Meningococcal | Mild strabismus |
| 6. | 7 months | *H. influenzae* | Major fits |
| 7. | 9 months | *H. influenzae* | Severe spasticity, blind Mental defective |
| 8. | 18 months | *H. influenzae* | Deafness |
| 9. | 2 years | *H. influenzae* | Deafness |
| 10. | 10 months | Pneumococcal | Spastic hemiplegia |
| 11. | 2 months | Unidentified | Spastic diplegia—mental defective |

TUBERCULOUS MENINGITIS (9 PATIENTS)

| | | |
|---|---|---|
| 1. | 7 years | Spastic hemiplegia |
| 2. | 4 years | Left facial paralysis |
| 3. | 6 months | Severe deafness |
| 4. | 4 years | Severe deafness |
| 5. | 7 years | Severe deafness |
| 6. | 9 years | Severe deafness |
| 7. | 12 years | Severe deafness |
| 8. | 5 years | Moderate deafness |
| 9. | 5 years | Slight deafness |

